# The wolf (canis lupus) as a symbol of an urban–rural divide? Results from a media discourse analysis on the human–wolf conflict in Germany

**DOI:** 10.1007/s00267-022-01719-3

**Published:** 2022-09-26

**Authors:** Jana Zscheischler, Jonathan Friedrich

**Affiliations:** 1 Leibniz Centre for Agricultural Landscape Research (ZALF), Eberswalder Str. 84, 15374 Müncheberg, Germany; 2 Department of Geography, Faculty II, University of Vechta, Driverstr. 22, 49377 Vechta, Germany; 3 Institute of Geography, Georg-August University, Goldschmidtstr. 5, 37077 Göttingen, Germany

**Keywords:** Environmental justice, Spatial justice, Wildlife conflict, Coexistence, Large carnivores, Rural populism

## Abstract

Given that wolves have been extinct in Germany for approximately 150 years, their return and growing population over the last decade has caused intense discussion and conflict. To develop a widely accepted and just coexistence between humans and wolves, a comprehensive understanding of the conflict is needed. There are indications that the conflict goes beyond dealing with the wolf population and marks a spatial–cultural divide between urban and rural areas. Nevertheless, the social dimensions of the human–wolf conflict in Germany have been little studied. The aim of this paper is to narrow this gap by means of a media discourse analysis including reader comments in order to provide insights into the constituent elements of this conflict. We conducted a qualitative content analysis. The sample comprises articles (*n* = 63) and reader comments (*n* = 515) over a period of one year (5/2018-5/2019) from six online periodicals in Germany. The results support the assumption of an urban–rural divide in terms of perspectives and values. The discourse indicates that rural actors who are most affected by the wolves’ vicinity have more negative attitudes towards them. At the same time, they feel abandoned and dominated by urban perspectives and politics. In addition, linkages to right-wing populist positions and conspiracy narratives that can be interpreted as a consequence of political alienation are found.

## Introduction

Worldwide, the recolonization of large carnivores such as wolves and bears often results in human–wildlife conflicts (e.g., Kinka and Young 2019; König et al. 2020). The case is no different in Germany. Gray wolves (Canis lupus) had been extinct in Germany for approximately 150 years, and their natural return from Poland and Eastern Europe to Germany and growing population over the last decade have caused intense discussion and conflict. From the perspective of nature conservation, the recolonization of gray wolves in Germany can be considered a great success. However, there are actors, particularly in rural areas, who perceive the return of the wolf as a threat and express concern about a reduction of their quality of life (van Eeden et al. 2021; Hamilton et al. 2020). In the 2018/2019 monitoring year, approximately 60 packs of wolves were counted in Germany. Although this wolf population size can still be regarded as very low, how to approach the wolf’s reemergence as a society has become debated in the highest political circles, and is the subject of extensive media coverage. Thus, the wolf population is featured almost daily in German media. A growing number of illegal killings have been recorded and indicate an escalating human‒wildlife conflict (see Yasmi et al. 2006; Frank 2016). It can be assumed that the resulting discourses and policy debates will also have implications for human‒wolf coexistence and conservation management (see van Eeden et al. 2021; König et al. 2020).

The human‒wolf conflict refers to perceived negative interactions between humans and wolves (Frank 2016). In particular, however, these are almost always social conflicts, meaning conflicts between humans over wolves (ibid.). Many scholars have emphasized that social conflicts can be understood as indicators of societal change, as drivers of development and transformation and that how we deal with conflicts relates to issues of justice (Boyer 2016), democracy, and recognition but also reveals social power relations (e.g., Gualini 2015; Mann and Jeanneaux 2009). Human–wolf conflicts therefore raise the question of how much space a society is willing to leave for not only the wolf population but also nature in general (Ojalammi and Blomley 2015). Furthermore, it is just as important to clarify to what extent the protection of the wolf population is democratically legitimized, which is related to issues of environmental justice and societal acceptance (e.g., Martin 2021). Consequently, we argue that achieving a better understanding of the human‒wolf conflict in Germany is important to develop well-accepted and equitable wolf management practices (see Ide 2016).

However, there are scholars who consider the focus on human‒wildlife conflict a constraint on finding successful conservation solutions and thus argue for broadening the perspective to include the concepts of coexistence and tolerance (see Frank 2016; Bruskotter and Wilson 2014). Coexistence between humans and wildlife can be viewed as the antithesis of the concept of conflict and as a general normative goal of wildlife conservation efforts. Frank (2016) considers the concepts of conflict and coexistence as two extreme points of a conflict‒coexistence continuum along which different actors’ attitudes lead to different levels of tolerances of wildlife. Overall, scholars argue that to develop successful conservation solutions and promote positive attitudes towards wildlife, it is important to explore and increase coexistence and tolerance rather than focusing on negative human‒wildlife interactions and thus conflict (e.g., Slagle and Bruskotter 2019; Frank 2016).

Increasingly however, findings suggest that human-wolf conflict points to overarching social tensions and broader conflict (e.g., van Eeden et al. 2021; Eriksson 2017). Thus, human intolerance of wolves often seems driven by conflicts between social groups, their different societal values, and conflicting views on environmentalism and ideologies (e.g., Slagle and Bruskotter 2019; van Eeden et al. 2021). In addition, there are nonmaterial costs of wolf conservation that have received less attention to date, such as fear, loss of cultural identity and local connectedness (Thondlana et al. 2020). These may lead to perceived injustices that interrelate with and amplify more fundamental social tensions. For example, Eriksson (2017) examined attitudes towards wolf policies in Sweden and found conflict to be a symptom of perceived powerlessness and political alienation. The human–wolf conflict is frequently associated with an urban–rural divide, indicating the perceived dominance of urban areas over rural ones (van Eeden et al. 2021; Højberg et al. 2017; Marchini 2014) and a perceived marginalization of rural actors (Dalerum 2021; Hochschild 2018; Marchini 2014; Theodorakea and von Essen 2016). Together, these results imply mistrust in public authorities—an assumption that is also supported by findings on different narratives of conspiracy (Skogen et al. 2022; Theodorakea and von Essen 2016) and attributed to phenomena such as postdemocracy (Crouch 2004).

Although some scholars feel that it is important to focus more on issues of tolerance and coexistence, we argue that focusing on conflict and relating discourses allows for the analysis of these broader, underlying social conflicts, which in turn will likely impact the achievement of successful coexistence. While there has been increasing research on human‒wolf conflict from the United States (e.g., Martin et al. 2019; Slagle et al. 2019; Walsh 2019; van Eeden et al. 2021) and Scandinavian countries (Bjerke et al. 1998; Ericsson and Heberlein 2003; Skogen et al. 2008; Eriksson 2017; Jacobsen and Linnell 2016) in recent decades, the results of these studies are based almost exclusively on quantitative studies and focus primarily on attitudes towards wolves and wolf management. In contrast, qualitative in-depth analyses of the human‒wolf conflict that seek a more comprehensive understanding of the conflict between social groups and underlying social tensions are still lacking (see also Figari and Skogen 2011). Moreover, there are still only few studies of the human‒wolf conflict in Germany (see Arbieu et al. 2019). This may be because the reemergence of the wolf population started later in Germany than in the Scandinavian regions, while in other parts of Europe, such as Spain and Italy, the wolf was never extirpated. However, Germany is a much more densely populated country, and wolves’ return may pose new challenges for coexistence. To date, the extent to which research results from the United States or Scandinavia can be transferred to these different socioecological and sociopolitical contexts has not been clarified.

Against this background, the aim of this study is to narrow this knowledge gap and gain a better understanding of the social conflict over wolves in Germany by means of a media discourse analysis. We follow the working thesis that conflicts surrounding the recolonization of wolves may reveal deeper underlying social tensions, such as the rural‒urban divide (cf. Kallert et al. 2021). Our study uses Germany as an example of a densely populated cultural landscape, with research thus far primarily focusing on sparsely populated areas. We use media discourse analysis as a window through which public discourse can be accessed (Boykoff 2008) and higher-level social conflicts become observable. The study is structured along the following three questions:

i. How is the human–wolf conflict in Germany constituted and documented in media discourse?
ii. Is the human–wolf conflict a symbol of the urban–rural divide?
iii. Why does the topic of wolf recolonization receive so much (media) attention?

## Discourses and socioenvironmental conflicts

Discourses are a significant sphere of society’s knowledge and meaning systems (Keller 2012). They take place on the micro level, in interactions between subjects, and on the macro level, as structured and formatted ways of debating specific topics. Discourses make a decisive contribution to the formation of social opinions of both subjects and collectives while at the same time reflecting such opinions (ibid.).

We refer to discourses as being defined as collections of objects, ideas, and concepts that mutually constitute stable meaning systems (Leipold et al. 2019). According to Foucault (2017), discourses constitute truth regimes and reflect power relations. This means that discourses can be seen as arenas in which truths and realities are defined by those in power (Keller 2007).

In addition to forming the collectively shared meanings of problems, discourses also provide subject positions that form the identities of individuals and social groups (Ide 2016). These identities are relational to “others against which a group defines itself” (ibid.). Based on identity construction and the resonance space for one’s own opinions and perceptions, discourses often shape social conflicts (Ashmore et al. 2001). Analysing discourses allows us to describe the content of these discourses, actors and their relations. Discourses are embodied in social practices by subjects in society and can thus lead to the design of material artefacts, that have a role in our everyday life. This mutually dependent relation has been highlighted by Fairclough (2013), who stated that discourses are material-semiotic at the same time and that these two aspects have “dialectic relations” (ibid., p. 9). This is based on the argument “that social realities have a reflexive character, i.e., the way people see and represent and interpret and conceptualize them is a part of these realities” (ibid. p.9). Media present one sphere in which public discourses become detectable and circulate (Van Dijk 1988). Mass media shape our “reality”, and journalistic discourses affect the composition of the everyday knowledge of a broad population (Luhmann 2000). Thus, media discourses can be regarded as a “resonance space” of societal discourses (Galanova and Sommer 2011) where collectively shared meanings, understandings and interpretations of problems and suitable solutions are (re)produced and transformed (Hajer and Versteeg 2005). Media can thus be considered a window through which the construction of specific knowledge circulation and opinion as well as emotions on specific issues can be analysed (Boykoff 2008).

Many studies have shown that media discourses are also representative of environmental discourses (e.g., Flaminio et al. 2021; Hopke 2012). Moreover, mass media can be considered a key stakeholder in conservation shaping social attitudes toward wildlife and wolves in particular (e.g., Arbieu et al. 2021). In the German media, regular reporting on the wolf population has formed a dense media discourse and thus allows comprehensive access to and observation of the related human–wolf conflict. In addition to articles in online and press media and their respective reader comments, there are fora on social media for intense discussion of wolf recolonization.

Against this backdrop, media discourse analysis was chosen as a suitable method to study the human–wolf conflict in Germany by focusing on sociocultural meaning structures as well as interrelations between different discourse elements and their role in the production of reality (Keller 2012).

## Methods and research design

### Data sample and selection

With the aim of capturing the discourse reflecting the human–wolf conflict in Germany, we analysed different online print media, including articles and user-generated reader comments. Different media have different target audiences, and to cover the broadest possible spectrum of the discourse, we selected media platforms by contrasting attributes describing their specific perspective: “urban” versus “rural”, “local” versus “national”, “tabloid” versus “key media”, and “pro-wolf” versus “anti-wolf”. These attributes were assigned to the media platforms after an initial screening and based on general knowledge about the German media landscape.

Table 1 presents an overview of the selected media and identified articles as well as reader comments used for the media analysis. We selected two daily local newspapers from the two regions in Germany where wolves have been experiencing dynamic population growth (Lusatia region and Lower Saxony), one specialist magazine in agriculture, two national leading media and Germany’s largest and most popular tabloid.

**Table 1:** Overview of the data sample: selected articles and reader comments

No.	Online print media	Description	Number of identified articles between 8 May 2018 and 7 May 2019	Number of selected and analysed articles	Number of selected reader comments
1	BILD	National tabloid; Germany’s largest and most popular daily	162	20	none^a^
2	TopAgrar	Specializing in agriculture with highest circulation in German-speaking countries (105,255 copies plus online access); monthly print edition complemented by daily news online	232	10	103
3	FAZ	National daily; bourgeois-conservative	28	10	250
4	Spiegel	National weekly; left-liberal	29	12	78
5	Lausitzer Rundschau	Local daily in area with dynamic wolf population growth	152	11	none^a^
6	Neue Osnabrücker Zeitung	Local daily in area with dynamic wolf population growth	42	10	131
	Total		645	63	515

^a^no reader comments available

All six online print media were scanned for articles relevant to wolf recolonization and published between 8 May 2018 and 7 May 2019. We identified 645 articles. As it was not possible to conduct a qualitative content analysis for such a high number of articles (*n* = 645), nor would it be useful with regard to the qualitative research approach, we reduced the number of articles for analysis. Qualitative approaches aim to show the range of different qualitative manifestations of a research subject and to theorize possible interrelationships and patterns. Determining the sample size and assessing its appropriateness therefore followed the frequently applied criteria in qualitative research studies of “theoretical saturation,” “informational power,” and “informational redundancy” (see Vasileiou et al. 2018). We initially limited the analysis to approximately 10 articles and the associated reader comments per media platform. We then successively involved further articles per medium in the analysis until no further insights (by additional codings) could be generated and, thus, “theoretical saturation” was reached. The final sample is described in Table 1.

The selection of these articles was guided by the following criteria:

i. The wolf (conflict) had to be a central topic of the article. Only mentioning it was not sufficient.
ii. The article must have focused on the normative dimension of the human‒wolf relationship in that meaning was attributed to this relationship. It did not matter whether the normativity came directly from the author or, for example, in the context of an interview or quotation.
iii. High numbers of reader comments attributed to an article were regarded as indicating high resonance by either favouring this perspective or holding antagonistic views. It can therefore be argued that these articles constitute good representations of discursive struggles, and they were preferentially included in our sample.

### Qualitative content analysis

Data processing was performed using MAXQDA software. All the articles and reader comments were evaluated and interpreted following the seven-step guide to qualitative content analysis by Kuckartz (2014) on the basis of an iterative research strategy using a deductive–inductive approach. This technique follows a consecutive and repeatable procedure that is based on the combination of a priori codes derived from the literature and the addition of inductive codes derived from the material. After the initial text work, including the writing of memoranda and case summaries, first, all the data were coded deductively by applying categories derived from a literature review on the human–wolf conflict (see Table 2; see steps 2–3 by Kuckartz 2014). Second, the category system was refined by adding additional inductive categories and subcategories derived from the material by in-vivo coding and paraphrasing (see steps 4–5 by Kuckartz 2014). This entailed an iterative process of rereading following the recommendations of Ryan and Bernard (2003; see also Bryman 2016), which also allows for explicit consideration of context, time, and space, as well as new findings from the empirics that have not yet been discussed in the a priori categories. Last, the first author of this article coded all the material with the developed coding system (see step 6 by Kuckartz 2014).

**Table 2:** A priori (deductive) category system derived from a literature review

Deductive category	References
Actors and their relationships (interests, identities, boundary markers)	Ide (2016); Zscheischler et al. (2019)
Urban–rural interrelations	Eriksson (2017); Theodorakea and von Essen (2016); Wilson (1997); van Eeden et al. 2021
Political alienation	Figari and Skogen (2011); Theodorakea and von Essen (2016)
Symbolic meaning	Figari and Skogen (2011); van Eeden et al. 2021
Attitude towards the wolf population	Bjerke et al. (1998); Eriksson (2017)
Conspiracy narratives	Skogen et al. (2008)
Conflict escalation levels	Højberg et al. (2017); Yasmi et al. (2006)

## Results

### Discourses in different media (general characteristics)

**Fig. 1:**
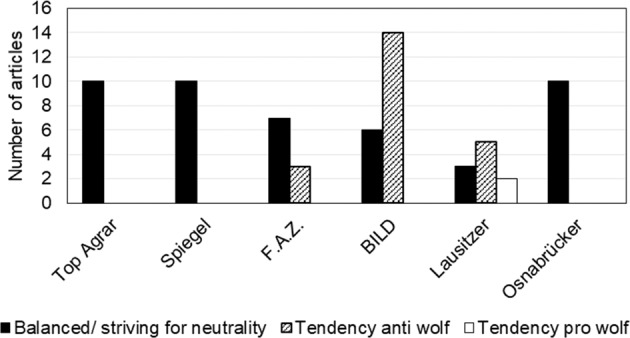
Tendency of reporting in articles by number

The results show varying discourse themes around wolves and variations in the frequency with which the different media cover wolves (see Tables 1 and 3). In general, the media report in a largely differentiated and balanced, factual manner (see Fig. 1). The exceptions to this are the conservative media companies FAZ and BILD. The conservative newspaper FAZ reports in a slightly biased manner, and some of its articles oppose the spread of the wolf population. The tabloid BILD reports in a strongly biased, dramatizing manner with a clear positioning against wolf recolonization. Below, we demonstrate that this contradicts the perception of some wolf opponents in the reader comments, who perceive the press as one-sidedly pro-wolf.

**Table 3:** Summary of discourse characteristics from the six selected and analysed media platforms

Media	General description	Main themes
Top_Agrar	As shown by the number of articles in Table 1, wolves are a top-priority issue. Coverage was almost daily during the investigated period. Most of the articles are written in a news style and rather short. While the articles cover different perspectives and aim at balanced and factual reporting, the reader comments are almost exclusively against wolf recolonization (100 of 103). All the analysed comments were written by 61 different readers, who were almost exclusively male.	Wolf management; wolf attacks and damage; population regulation; conflict
Spiegel	All the articles are formulated in a factual style. Wolves are consistently presented as a species that is in danger of extinction in Germany and poses a low risk to humans. Two of ten articles refer to the AFD’s (right-wing populist and extremist party in Germany) use of the subject to win votes. In addition, emotionalization of the debate is juxtaposed with its practical relevance. The reader comments are a combination of pro- and anti- wolf statements. The gender of the commentators cannot be clearly deduced, as pseudonyms are used.	Wolf as a political issue; damage; wolf attacks; knowledge; conflict; trade-offs/ goal conflicts with nature conservation
FAZ	The majority of the articles analysed are concerned with factual debate (*n* = 7). The most tendentious articles (*n* = 3) present the perspective of those opposed to wolf recolonization. Most of the reader comments are very critical of or negative about the return of the wolf population. Here, too, the commenters are predominantly male.	Wolf dispersal; conflict and politicization; goal conflicts; damage and cost; regulation of wolf population
BILD	After TopAgrar, BILD has the most articles on wolves among national media companies. Among the 20 analysed articles, factual news-style reports can also be found, but these are rather the exception (6 of 20). Overall, the reporting is tendentious and dramatizing and appeals to readers’ emotions (e.g., fear). Conservative perspectives (e.g., of farmers, hunting associations, the CDU) are most common.	Damage and risks stemming from wolf population growth and recolonization; changes in German hunting law; wolf attacks; illegal wolf killing; wolf management; goal conflicts with species protection
Lausitzer Rundschau	For the most part, the reporting is objective; nevertheless, there is a slight emphasis on the views of those opposed to wolf resettlement: neutral presentation, *n* = 3; pro-wolf, *n* = 2; and tendentious and anti-wolf, *n* = 5.	Changes in German hunting law; wolf population development; wolf management
Neue Osnabrücker Zeitung	There is factual and balanced reporting throughout that presents different perspectives (e.g., nature conservation, farmers’ associations, hunters, politicians).	Conflict; wolf management; increasing population; illegal wolf killing

**Fig. 2:**
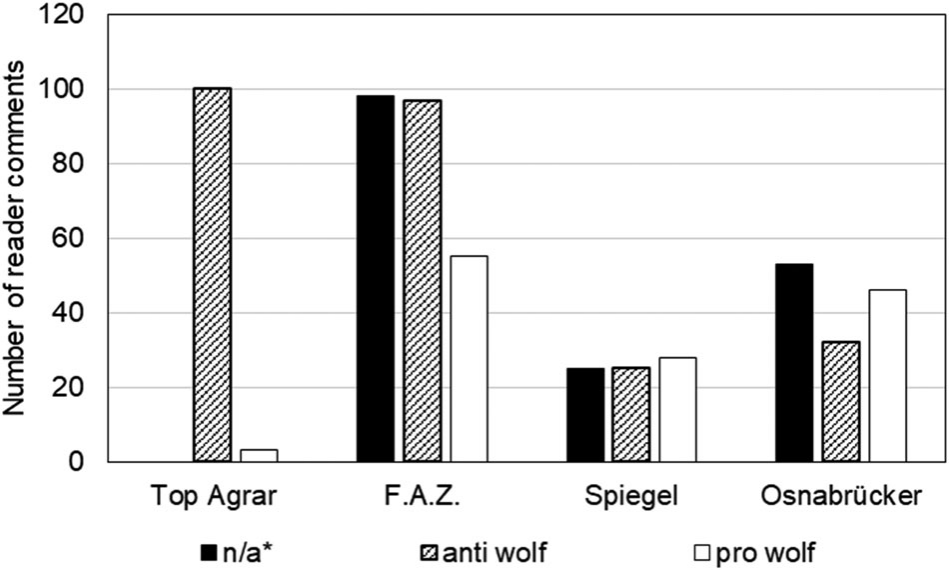
Perspectives of reader comments in terms of number of comments. *Not all the reader comments could be clearly categorized because they included sarcastic or unclear statements unrelated to the topic of the wolf population

In the media reports, no positions completely reject the idea of wolf resettlement, indicating basic tolerance for passive coexistence. Rather, the discourse revolves around questions of appropriate wolf population management, which could be important conditions for acceptance. This includes questions of population regulation, changes in hunting laws, damage that has been caused and corresponding compensation measures. Compared with the contributions of the media, the reader comments are less differentiated, and most can be considered completely pro- or anti-wolf (see Fig. 2).

### Main themes in the discourse on wolf resettlement

The themes that appeared most frequently in the analysed material were: (1) actor groups and their mutual relations or perspectives/perceptions, including conflict over interpretative sovereignty; (2) an instrumental utility discourse about benefits versus costs, risks and goal conflicts connected with wolf resettlement; (3) an urban–rural divide and political alienation; (4) links to right-wing populist discourses, narratives of conspiracy and forms of escalation; and (5) issues in wolf management. Table 4 provides an overview of the evolved coding hierarchy and the content structure of the analysed material. In the following, we will describe these five main themes in more detail. The results are illustrated by exemplary quotations from the media articles and reader comments either directly in the following text or with reference to quotations in the Supplementary Material of this article (Qn, for an overview of all the quotations, see Supplementary Material).

**Table 4:** Coding system of deductive and inductive (sub) codes developed by qualitative content analysis (after Kuckartz 2014)

Main themes	Categories	Subcategories/ “in vitro” coding (selection)
1 Actors’ relationships and shared perspectives		
Actors	Agriculture	Pastoralists; shepherds; livestock farmers; Farmers’ Union;“Extreme farming destroys nature”; “Farmers are whiners”; “Farmers are being used as scapegoats”
	Hunters	
	Rural people	
	Nature conservation	NGOs (NABU and WWF); “nature conservation radicals”; “green wolf romantics”; “wolf freaks”; “wolf protectors”; “unscrupulous animal rights activists”
	Politicians	Politicians lack expertise; politicians are inactive; politicians have no grip on reality; parliament determines wolf population policies; nature conservation is an “elite project”
	Urban people	“Urban elite”; “ecobese urbanites”; “wolf metropolists”
	Scientists and experts	So-called experts
	Media	Press is biased and “pro-wolf”; press stirs up panic and hysteria; media are controlled by NABU
Conflict about interpretative sovereignty (“Deutungshoheit”)	Devaluation of wolf opponents (by attributing to them a lack of expertise and credibility) Devaluation of wolf proponents (by attributing to them a lack of expertise and credibility)	Irrational fears; “hysterical cries fear”; scaremongering; unobjective discussion “Eco/wolf romantics”; “eco-social romantic fairy tale ideas”; “wolf freaks”; wolf advocates lie; ignorance and bossiness
2 Discourse on benefits versus costs		
Damage, costs and risks caused by wolves	Diminished quality of life in rural areas Killed livestock Costs of prevention measures and taxpayers’ money Lack of benefits	Fear; wolf is a risk and dangerous to people and children; limited playground area for children Wolves present little danger; life itself is risky; dogs are more dangerous Compensation costs; emotional damage “Wolves are a luxury”
Benefits/Functions of the wolf	Instrumental utility Intrinsic value	Reestablish ecological balance; tourist attraction “Predators belong in our nature”; “man rapes nature and makes everything subservient”
Target conflicts	Conservation	Extensive pasture grazing; cultural landscapes versus natural landscapes; animal welfare of killed livestock
3 Urban–rural divide	Perception of rural people	“Urban elite” live in “another country”;
	Spatial injustice Political alienation	urban majorities in parliament; dominance over rural people;
		“Wolf policy clearly directed against the rural population”; rural areas disadvantaged; urban people who are not affected want the wolf to remain
	Berlin/Brussels as centres of political power	Decisions are made remotely
4 Antidemocratic positions		
Right-wing populism	Wolf resettlement as an “AFD acceleration programme”	Wolves are an election campaign issue
	Right-wing populist language and references to refugee crisis in 2015/2016	“Lying press”; “Willkommenskultur”; “upper limit”
Conspiracy narratives	Wolves are hybrids and brought to Germany Concealment of information Scientists falsify results NGOs want to make money	
Conflict escalation	Emotions and hardened positions Protest and campaigning Illegal wolf killing Calls for criminal action	
5 Wolf management	Wolf population control/stock regulation	Moderate population regulation; sharp hunting; protection hunting; upper limit; shooting in case of danger; population regulation important for acceptance
	Herd protection	Crushing; fences; guard dogs; livestock management must adapt
	Legislative changes	Hunting law; designate wolf (and wolf-free) areas; prohibit wolf feeding; facilitate extraction
	Compensation payments	Fair compensation
	Conflict management	Improved Information and knowledge; wolf commissioner; training and education; balancing interests and increasing acceptance

#### Actor groups and their relationships/mutual perspectives

Actors with opposing values, perspectives and interests are a central part and constituting category of the “human–wolf” conflict in Germany, which largely indicates a social conflict over wolves. The results show that views in the conflict about wolf resettlement in Germany are characterized by two strongly opposed dichotomies:

i. agriculture versus nature conservation and
ii. rural people versus the “urban elite”.

In addition, there are distinct actor groups, such as politicians, scientists and experts, hunters and the media, forming or supporting the parties in the conflict. In the content studied here, these positionings resulted from attribution of criticism and devaluation of opposing positions. It was apparent that the conflict over the reintroduction of wolves is also a conflict over interpretative sovereignty. Frequently, strategies were used to devalue opposing arguments and strengthen the actor’s own position. Credibility, expertise and knowledge became contested attributes denied to the other side through degrading attributions. These devaluation mechanisms were used by wolf supporters and wolf opponents alike but with different attributions and levels of intensity. Thus, references to other social groups of actors came primarily from opponents of the wolf-recolonization efforts. From the perspective of many wolf-resettlement opponents, nature conservationists and urban people were “wolf romanticists” and “wolf freaks” with “eco-social romantic fairy tale ideas” who are “far removed from reality”. Further attributions included that they are “ignorant and [have a] know-it-all attitude”, they enforce “double standards” and tell “lies”, they “lack expertise”, and they engage in “unobjective/ideological/emotional discussions”.

Opponents of wolf recolonization considered politicians to lack a firm “grip on reality” and “expertise” and to be “idle/actionless” while practising politics only for the benefit of the “urban elite”. Some wolf-recolonization opponents accused the media of being biased and “pro-wolf”. However, the analysis of the positioning of the articles studied contradicted this accusation (see 3.1), as it showed that media coverage of the wolf population is varied and not one-sided.

In contrast, some proponents of wolf resettlement accused those opposed of “scaremongering”, having “irrational fears”, exhibiting “hysteria”, and engaging in an “emotional debate”. Farmers were considered “complainers” who destroy nature with their agricultural practices.

#### Discourse about benefits versus costs, risks and target conflicts

Another main theme throughout the media articles and reader comments was a benefit-versus-cost discourse. The “damage” caused by wolves was frequently used as an argument against wolf resettlement. Often mentioned, damage included “livestock killed by the wolves”, potential “risks to people and especially to children”, “fear of wolf attacks” and a resulting “diminished quality of life” due to the perceived restricted range of motion for humans and playground areas for children. In addition, the cost of prevention measures funded by taxpayer money was criticized. Target conflicts in nature conservation were cited, particularly regarding the high nature value of extensive pastureland, which is regarded as endangered by wolf recolonization. A further ethical conflict was seen regarding animal welfare when wolves kill grazing animals in fenced pastureland.

In addition to the discourse about damage, the general benefits of having wolves were questioned by wolf-recolonization opponents (Q1; for an overview of all the quotes, see Supplementary Material). In particular, wolf recolonization in Germany’s densely populated “cultural landscape” was repeatedly denounced (Q2–4). Here, the statements documented the perception of or fear of competition with wolves and the narrowing of humans’ interests and scope for action:

Q2: “But here, where every German citizen has only 2000 square metres of usable space to ‘survive’, one should differentiate a little as to who is allowed to sit at the table. The wolf does not fit here.” (Reader_TopAgrar)

Strikingly, the wording of comments frequently implied active settlement by wolves instead of a natural dispersal process (Q3).

Reader comments also included arguments for the protection of species of larger predators in distant regions of the world but not in their own immediate vicinity (Q4). This form of colonization of other countries to further species conservation was considered critical by some wolf-recolonization advocates, who noted that the conservation of large carnivores and mammals in countries in the Global South is more precarious (Q5-6).

Q6:“If a tiger is shot in India, the German is the first to raise a finger. But we don’t care about our own fauna.” (Reader_Spiegel)

While some saw the wolf as an unnecessary “luxury” that causes great financial damage, which must be remedied by adequate compensation, a main argument against wolf dispersal was the perceived fear and risk of humans being attacked by wolves (Q7-8), especially the danger that wolves present to children, which was a recurring theme in the discourse (Q9). This fear, particularly of the danger posed to children by wolves, was a motif that ran throughout the reader comments (Q10-11). In this context, the feeling of not being taken seriously was also found.

Q12: “As you often hear, the fears you describe exist, whether justifiably or not is irrelevant. I ask myself why we want to do this to ourselves, or to those who are afraid, just to prove a few opinionated wolf romantics right. Fear is not a nice thing, and one should not make fun of it.” (Reader_NOZ)

In contrast, the views and arguments expressed by supporters of and experts in wolf resettlement revealed that compared with other everyday risks, they viewed the risk as very low or acceptable. Compared with wolf-recolonization opponents, who focused on the uncertainty and potential risk posed by future developments, wolf-recolonization supporters and experts highlighted that there have been no fatal wolf attacks on humans thus far (Q13). Other arguments emphasized that humans constantly live with certain risks (Q14). In addition, proponents of wolf recolonization argued for the potential benefits of wolves as a regulating force against game browsing and damage to crops. They considered the wolf to play an important role in “reestablishing the ecological balance”. In the Lusatian region, a main habitation area for wolves in Germany, tourism experts even believe that the wolf population has the potential to become a tourist attraction (Q15).

Beyond a pure cost–benefit argumentation that considers the wolf merely from an instrumental utility perspective, there were also reader opinions that attributed an intrinsic value to the wolf. These actors criticized the colonial dominance of humans and recognized the right of the wolf as a species to exist for its own sake. However, this last argument was rarely put forward.

#### Urban‒rural divide and political alienation

The reader comments documented contrasting perceptions of the realities of life in the city and the country, coupled with processes of political alienation. The discourse painted a picture of disparity between city and country, between an “urban elite” and the rural population (Q16). There was a perceived political “dominance by urban people” and their opinions over the rural population. At the same time, the rural population was portrayed as an oppressed minority (Q17–18).

In addition, the difference in the degree to which urban and rural populations are affected by wolf recolonization was emphasized by some readers’ comments. The support for wolf resettlement found among urban people, who are rarely affected by the presence of the wolves, was perceived as arrogance towards the rural population (Q19–22).

Q19: “The people in the areas where wolves live are already suffering from massive restrictions on their quality of life. […] It’s unfair for nature-minded urban people to be in favour of the wolf, which they will never encounter in their lives!” (Reader_FAZ)

Q20: “We are bombarded with wolf propaganda by the nature conservation authorities. But when concerned graziers show the cruel reality, the state authorities intervene. What a complete dismissal of the rural population!” (Reader_TopAgrar)

Here, a motif of perceived spatial injustice emerges, which was also associated/contextualized with other developments and conflicts in rural areas, such as the energy transition and the conflictual designation of areas for wind turbines (Q23) or the growing demand for organic food and sustainable agricultural practices, which increasingly pressures farmers to change (Q24). Urban people were accused of lacking an understanding of nature, being out of touch with reality, and lacking judgement (Q25–27). The city of Berlin recurred not only as a symbol of urban living and as an urban space but also, alongside the city of Brussels, as a political centre of power where decisions about rural areas are made removed from the context in which the issues occur (“in distant Berlin”) (Q28–29). Mixed into this was also a demonstrable loss of trust in politics and the state, feelings of political alienation and sympathy with populist positions (Q28, Q30–32). Politicians were frequently accused of inaction, but the opposite was found in the media coverage. Despite the many other challenges brought by development in rural regions, such as demographic change, the wolf population received special attention and played a comparatively large role in both the media and politics (Q33). Thus, the wolf recolonization conflict was discussed at the highest political levels during the study period, including by the Chancellery, the Prime Minister of Saxony, and the Ministers of the Environment and Agriculture.

#### Linkages to right-wing populist discourses, narratives of conspiracy and forms of escalation

The political alienation that was evident in the analysed material was regularly linked to sympathy for right-wing populists and antidemocratic positions (Q30–32). In the discourse on the wolf population, vocabulary typically used by right-wing populist groups appeared conspicuously often. The use was documented exclusively in reader comments by those opposed to wolf recolonization in Germany. In addition to the motif of the “lying press” (Q34-35), there were recurring allusions to “conspiracies” by “elites” and rejecting the European Union, and references to the 2015/2016 refugee crisis in Germany and Europe were made using the term “Willkommenskultur” (Q36), which was influential at that time.

It was not just the reader comments that took up this issue; the articles themselves examined it as well. The articles revealed that defensiveness about wolf recolonization is being made an election campaign issue by the right-wing populist party AFD in Germany (Q37–38). In fact, the Federal Minister of Agriculture considered the wolf an “AFD acceleration programme”, as anti-wolf positions were strongly represented by and related to the AFD party.

In addition, various conspiracy narratives were found in the reader comments. One common narrative was that the wolves are hybrids that were deliberately brought to Germany and released. Another narrative asserted that important information was being kept secret (Q39). It alleged a conspiracy among scientists, politicians and actors from NGOs: scientists were purportedly falsifying results in their expert reports, and NGOs would earn money from the wolves.

The media discourse on wolves is divisive and polarizing, and there are signs of increasing escalation and intensity in the conflict, which manifest in hostility and threats. Not only emotional statements and mutual accusations but also insults were observed in exchanges between the parties in the conflict, and these pointed to hardened positions (Q40). In addition, various forms of protest, such as vigils, demonstrations and social media campaigns, as well as calls for “illegal wolf killing”, were mentioned. This call for criminal action was justified as a form of self-defence (Q41–43).

#### Wolf management issues

One of the most frequently discussed topics were wolf management issues. These included “stock regulation/wolf population control”, subsequent demands for “legislative changes”, aspects of “herd protection” and “compensation payments”, demands for “conflict management” and “counselling and information services”, such as training and education to increase public knowledge about wolves.

Overall, in concrete discussions of suitable wolf management measures, the discourse notably took on a more objective tone. Emotional or sarcastic expressions were less commonly observed. The positions expressed seemed more conciliatory and solution-oriented, and the conflict appeared less intense in such discussions.

Regarding population regulation, there were very few voices in favour of extirpating the wolf completely, but many favoured population regulations. Opinions ranged from allowing “strict population regulation/sharp hunting” (Q44–45), setting an “upper limit” on moderate regulation and removing wolves “in case of danger” or “problem wolves”. The latter is also seen and advocated as a measure to promote acceptance of the wolf population (Q46). The demands for wolf population regulation went hand in hand with demands for legislative changes that allow the removal of wolves as well as the designation of wolf-free or protected grazing areas. In addition, herd protection measures and the adaptation of pasture management, including installing fences, stable security, using guard dogs and scaring away wolves, were discussed. Commenters who had already had good experiences with these measures also spoke up here. There were several indications that damage from wolves mainly occurs where there is no or minimal herd protection.

There were many reader comments that demanded comprehensive “compensation payments” for the herd protection measures enacted and the damage caused by wolves. However, this appears to be somewhat irritating, as 100% of these costs are already being reimbursed. In this context, it seems natural that the idea of “conflict management” was often alluded to in the articles (Q47) in reference to learning processes, training and information services that could increase acceptance.

## Discussion

The aim of this article was to analyse the human‒wolf conflict in German media discourse, determining how the conflict is constituted and documented, whether it is a symbol of an urban‒rural divide, and why the topic is covered so intensively in the media. In the following, we will discuss our findings in relation to results from other geographical contexts (5.1) and justify our argument that the human‒wolf conflict is indicative of broader societal conflicts, such as the urban‒rural divide (5.2).

### Human–wolf conflict in Germany and elsewhere

Our findings about the human–wolf conflict in Germany have much in common with the results of studies in other regions of Europe and North America. The return for wolves in regions where they have been previously extinct creates conflicts (e.g., Ericsson and Heberlein 2003; Bisi et al. 2010). Spatial proximity and affectedness have been shown to have a crucial influence on attitudes towards wolves (see Eriksson 2017; Dressel et al. 2015; Slagle et al. 2019). Although the reader comments analysed here cannot be clearly attributed to urban or rural actors, differences between the attitudes of actors in rural and urban areas are clearly indicated. Thus, narratives of “pro-wolf” urbanites and “anti-wolf” ruralites dominate the media discourse.

Dressel et al. (2015) have shown that people who have been harmed by wolves, such as farmers or hunters, are less positive about the reintroduction of wolves. Not surprisingly, this finding can be supported by the present study. Damage caused by wolves, such as killed livestock and high costs for preventative measures, are a major topic in the German discourse on this subject. Aggrieved parties remain vehemently against wolf recolonization despite the extensive compensation measures put in place, indicating that the nonmaterial costs of recolonization also play an important role (Thondlana et al. 2020). Thus, the perception of a reduced quality of life due to fear and perceived risks caused by wolves plays a major role in the debate. Wolf advocates and conservationists never tire of noting that since the year 2000 - when wolves returned to Germany - there have been no documented instances of aggression by wolves toward humans. However, communication approaches of this kind will most likely remain ineffective because, as is well known from risk theory, perceived fear and risks are not the result of logical analysis. Rather, they are a function of social trust in managing authorities and perceived control over wildlife hazards (Bruskotter and Wilson 2014).

Our analysis further reveals a prevailingly utilitarian discourse in the German media that questions the wolf against the background of a cost–benefit discourse. The wolf is seen as “useless” and harmful by farmers, and its presence thus deepens the already existing conflict between stakeholders in agriculture and in nature conservation (see also Friedrich et al. 2021). However, there are also conflicts within nature conservation. Since the wolf makes grazing livestock - an important tool for conservation management - more difficult, a conflict of objectives arises between species conservation on the one hand and the preservation of cultural landscapes with high levels of biodiversity and natural value on the other.

The observation by Figari and Skogen (2011) that the wolf represents a kind of antithesis to civilization cannot be fully supported by the results of this study. Rather, it seems that there are different ideas and expectations for how human–nature relations should be shaped. Thus, the cultural landscape is dichotomously set against the wilderness and is described by opponents of wolf resettlement as an unsuitable area for the coexistence with wolves.

All the media investigating the wolf topic continually reported on it. The issue received significant attention, and the debate can be regarded as emotionalized. A similar level of attention was also observed in Northern European countries after wolf populations began to spread again (Ericsson and Heberlein 2003), while there were fewer studies in Southern and Eastern European countries where the wolf was never extirpated. Chapron and López‐Bao (2020) attribute this to cultural differences in dealing with dissent or conflict and argue that conflict is more socially accepted in Mediterranean countries. They support this assertion by pointing out the lower amount of scholarly attention to the human–wolf conflict in these countries and the resulting lack of conflict studies there. However, until now, there have also been hardly any studies on the human–wolf conflict in Germany. Importantly, the results clearly show that conflict exists and is intensifying. Nevertheless, the wolf is a relatively new phenomenon in Germany that requires adaptation by some actors, therefore producing resistance. The observed conflict is thus a reaction to change (Bisi et al. 2010; Wilson 1997). While the wolf was never eradicated in some Southern European countries, it has been able to spread again in Northern Europe only in the past two to three decades. Thus, in areas where wolves were once extinct, their return is particularly controversial (Chapron et al. 2014). Consequently, it is conceivable that the conflict will be defused over time through habituation effects among rural residents but also through learning and adaptation processes in animal husbandry grazing practices. However, there is not enough evidence to support this assumption. To date, few studies have examined the course of such conflicts and there is a major knowledge gap in their handling or management.

### Wolf as a symbol of the urban–rural divide

Consistent with Wilson’s (1997) observation that wolves “are merely symbols delineating the battle of a much larger conflict” (p.453), the results of the current study show the clear wider significance of the human–wolf conflict. The reemergence of wolves in Germany represents a larger social conflict regarding the shaping of human–nature relations. In this context, several authors of distinct studies in different regions reveal that the wolf symbolizes different identities within the sociocultural urban–rural divide (e.g., Figari and Skogen 2011; Theodorakea and von Essen 2016; van Eeden et al. 2021). The same pattern emerges from the results of the conflict analysis for Germany presented here.

Moreover, these results show that the wolf symbolizes the political alienation of the rural population, who feel dominated by urban worldviews influencing policies and the will of supposed majorities. Eriksson (2017) showed that attitudes towards the wolf are particularly affected by such political alienation. This may be because while rural areas are most impacted by the return of the wolf, urban areas can welcome its reintroduction without having to fear the negative consequences (see also Jordan et al. 2020). This raises fundamental questions of spatial justice (e.g., Barraclough 2013; Davoudi 2013) and may harden already-existing conflicts. At the same time, the rural population seems to project whatever is normatively unwanted onto so-called “urban elites”, thereby also constructing their own identity as a reaction to feeling “unheard”. While the results document the concern of some rural stakeholders that wolves could be dangerous and reduce humans’ perceived security, this concern is dismissed as unfounded by wolf supporters, especially wolf experts. This contrast reflects a feeling among rural stakeholders that their fears and concerns are not being taken seriously enough by decision-makers (see also Thondlana et al. 2020).

Additionally, some scientists, especially ecologists normatively involved in conservation, seem dismissive of these concerns. As an example, Chapron and López‐Bao (2020) criticize the nature of social science research on conservation conflicts regarding large carnivores. In their view, seeking consensus and adopting an anthropocentric view would soften conservation and subordinate it to human needs. According to these authors, the most important actor – nature - remains unheard in such a practice. However, the literature on environmental justice shows that there is not only an either/or choice between “anthropocentrism” or “ecocentrism” but also relational valuing of both nature and humans (cf. Martin 2021). Even if the view of Chapron and Lòpez-Bao (2020) is logical, the argument remains that the costs and benefits of human coexistence with wolves are unequally distributed. Jordan et al. (2020) called this phenomenon “coexistence inequalit[y]” and pointed out that the costs of coexisting with wildlife (particularly large carnivores) are predominantly borne by the Global South and rural communities, while the benefits go to the Global North and urban residents. The results of the present study confirm this. However, they also show that while advocates for wolves do reflect on these “coexistence inequalities” between the Global North and the Global South and use them in their arguments, they rarely address the disparities between urban and rural areas. Jordan et al.’s (2020, p. 807) suggestion of developing empathetic advocacy in terms of “working for wildlife while demonstrating empathy for those bearing the costs of human–wildlife conflicts”, we argue, should receive further attention from scientists and politicians alike.

Nevertheless, the geographical and spatial disparities between urban and rural areas have led to increasing social distortions in recent years, which are also reflected in the successes of right-wing populist parties (e.g., Kallert et al. 2021; Rodríguez-Pose 2018) and becoming visible in other areas, such as energy transitions and land-use conflicts (Fienitz and Siebert 2021). These parties like to seize upon the problems and frustrations of those living in rural areas to turn them against “urban elites” and project problems onto this diffuse group (Deppisch et al. 2021; Hochschild 2018). The results of this study provide evidence of this mechanism. However, they also clearly reveal that reader comments exhibit a predominantly binary set of views on this issue—either pro- or anti-wolf. Moderate voices that demonstrate mutual understanding and empathy are rarely found. Hochschild (ibid.) calls this an “empathy wall” and sees the lack of mutual understanding and polarization as a hurdle to future democratic debate. In this context, our findings also show that political alienation is often accompanied by right-wing narratives and conspiracy theories (see also Marchini, 2014).

However, further research is needed to elaborate whether the conflict line is the rural‒urban divide or whether other conflict lines are congruent with this line; thus, other sociocultural aspects may be relevant, as described in recent theoretical and empirical sociological research in Germany (e.g., Lux et al. 2022; Reckwitz 2021).

In Europe, wolf recolonization is a result of, and supported by, international nature conservation law, such as the Washington and Berne Convention and the EU Habitats Directive. However, this is a top-down approach that insufficiently considers local specificities and region-specific development opportunities in rural areas. The wolf and its coexistence with humans thus seem politically imposed, which inhibits successful coexistence (König et al. 2020). In our view, an important goal of conservation management should be to increase tolerance of the wolf among the rural population. However, economic compensation measures alone are considered insufficient; they ultimately represent a purely technical response in theories of environmental justice (e.g., Martin 2021) and do not address the nonmaterial costs of wildlife conservation (Thondlana et al. 2020). Our results show that different conceptualizations of nature–human relations become explicit in the discourse surrounding wolves in Germany, which underlines the findings of Jacobsen and Linnell (2016) in that environmental justice (based on human-nature relations and values) is understood in different ways. These different understandings are a major barrier to coexistence between carnivores and humans that is perceived as just and fair. It is therefore necessary to strengthen institutional justice and to design processes that are perceived as legitimate and just by all stakeholders. We argue that ongoing dialogue is needed in which the interests of rural stakeholders are heard and adequately included in decisions regarding wolf management. This refers to the role of “recognition” frequently emphasized in the literature on environmental justice (see Coolsaet 2020). The involvement of local stakeholders could support mutual learning about and co-design of well-accepted wolf management measures, increase knowledge and thus perceived control over wolves, develop “empathetic advocacy” on the part of conservationists and finally promote trust in management authorities, which are all frequently emphasized preconditions to increase tolerance of wildlife (e.g., Bruskotter and Wilson 2014).

### Methodological reflection and limitations

To date, human‒wolf conflict in Germany remained understudied. In particular, qualitative analyses that allow for an integrative understanding are lacking. The present media analysis contributes towards filling this gap. Overall, it shows that media analyses achieve considerable density in the qualitative description of human‒wildlife conflicts. However, this description takes place on a superordinate aggregated societal level, which can differ greatly from local specific and varying manifestations. In addition, media filter information according to newsworthiness and also attention or entertainment value. However, even the reader comments analysed here do not provide a representative depiction of the human‒wolf conflict but rather indicate the different dimensions and facets that play a role. For a deeper understanding of the conditions for successful coexistence, therefore, further analyses are needed that look at different local characteristics and also at the interactions of different social factors with varying management instruments and communication approaches.

## Conclusion

The recolonization of wolves in densely populated cultural landscapes causes major social conflicts reflected by a high amount of media attention. There is a divide in the perspectives of urban and rural actors, which is supported by the narrative of “pro-wolf” urbanites and “anti-wolf” ruralites and is also visible in the media coverage. The unequal distribution of the nonmaterial costs and benefits of the recolonization of the wolf population results in “coexistence inequalities”, which in turn may lead to political alienation and a sense of imposition. However, the human–wolf conflict symbolizes a larger conflict based on spatial disparities and inequalities between urban and rural areas. Damage prevention and compensation measures alone will not be sufficient to resolve the conflict. Going forward, it will be important to consider regionally specific development options, strengthen local decision-making competences and facilitate honest participation and dialogue so that different actors with different opinions are valued in decision-making. Only in this way can a just and sustainable coexistence between humans and wolves be established.

## Supplementary information

Supplementary Information
