# Supplementary material for: The wolf (canis lupus) as a symbol of an urban–rural divide? Results from a media discourse analysis on the human–wolf conflict in Germany
Source: Environ Manage. 2022 Sep 26;70(6):1051–65. doi: 10.1007/s00267-022-01719-3 (PMC9622530; doi:10.1007/s00267-022-01719-3)
Supplement: Supplementary file 1 — Supplementary Information [file 267_2022_1719_MOESM1_ESM.docx]

**Supplementary Information: Quotes from articles and reader comments**

| **Qn** | **Quotes from articles and reader’s comments – German Original** | **Quotes from articles and reader’s comments – German Original – English Translation** |
| --- | --- | --- |
| Q1 | *“Ohne Zweifel sind Natur und Kultur(landschaft) seit über hundert Jahren ohne Wolf gut ausgekommen. Die Rückkehr des Wolfes in eine jetzt völlig anders strukturierte Umgebung stiftet vernehmbare Unruhe und bringt keinen erkennbaren Nutzen”. (Reader_FAZ)* | *"There is no doubt that nature and culture (landscape) have managed well without wolves for over a hundred years. The return of the wolf to a now completely differently structured environment causes clear unrest and brings no discernible benefit". (Reader_FAZ)* |
| Q2 | *"Aber hier wo jeder Bundesbürger nur 2000 qm Nutzfläche zum "Überleben" hat, sollte man doch etwas differenzieren, wer mit am Tisch sitzen darf. Der Wolf passt hier nicht her…”* (Reader_TopAgrar) | *"But here, where every German citizen has only 2000 square metres of usable space to ‘survive’, one should differentiate a little as to who is allowed to sit at the table. The wolf does not fit here". (Reader_TopAgrar)* |
| Q3 | *"Deutschland gehört mit 233 Einw. pro qkm zu den am dichtesten besiedelten Ländern in der EU. Dass ausgerechnet hier der Wolf - koste es was es wolle - flächendeckend und dauerhaft angesiedelt werden soll, ist nicht nachvollziehbar!”* (Reader_FAZ) | *"With 233 inhabitants per square kilometre, Germany is one of the most densely populated countries in the EU. It is incomprehensible that here, of all places, the wolf should be permanently settled—at any cost!" (Reader_FAZ)* |
| Q4 | "Es hatte (triftige) Gründe, dass der Wolf in unserer dicht besiedelten Landschaft ehemals beseitigt wurde. In Sibirien gibt es Wölfe genug, bei uns ist er fehl am Platze”. (Reader_FAZ) | "There were (good) reasons why the wolf was formerly eliminated from our densely populated landscape. In Siberia there are wolves enough, with us, it is out of place". (Reader_FAZ) |
| Q5 | *"Wenn wir den Wolf wieder abschaffen wollen, müssen wir anderen auch erlauben Löwen, tiger und Elefanten auszurotten, die große wirtschaftkiche Schäden in deutlich prekären Lebensverhältnissen anrichten, die nur selten ausgeglichen werden”. (Reader_FAZ)* | *"If we want to abolish the wolf again, we must also allow others to exterminate lions, tigers and elephants, which cause great economic damage in clearly precarious living conditions that are only rarely compensated for". (Reader_FAZ)* |
| Q6 | *"Wird in Indien ein Tiger erschossen, ist der Deutsche der erste, der den Zeigefinger erhebt. Aber unsere eigene Fauna ist uns egal..". (Reader_Spiegel)* | *"If a tiger is shot in India, the German is the first to raise a finger. But we don't care about our own fauna". (Reader_Spiegel)* |
| Q7 | *"Eine Frau aus dem Dorf erzählt, dass ihre Tochter sich nicht mehr im Dunkeln zur Bushaltestelle traue”. (Artikel_Spiegel)* | *"A woman from the village says that her daughter no longer dares to go to the bus stop in the dark". (Article_Spiegel)* |
| Q8 | *"Ihre Konsequenzen aus den realen und vermuteten Wolfssichtungen um Hohenleipisch hat eine Frau aus einem der Eigenheime am Rande des Dorfes gezogen: "Abends gehe ich nicht mehr raus”. (Artikel_LZ)* | *"A woman from one of the homes on the edge of the village has drawn her conclusions from the real and suspected wolf sightings around Hohenleipisch: ‘I don't go out in the evenings any more’". (Article_LZ)* |
| Q10 | *"Diese Tiere sind Raubtiere...sie suchen sich die Opfer die am leichtesten zu erbeuten sind....ob das Schafe, Rehe, Ziegen, Hasen oder kleine Kinder sind”. (Reader_Spiegel)* | *"These animals are predators...they seek out the victims that are easiest to prey on...whether that be sheep, deer, goats, rabbits or small children". (Reader_Spiegel)* |
| Q11 | "Okay, warten wir, das erledigt sich von selbst. Das nimmt von allein Geschwindigkeit auf und wird irgendwann zu einem Super-GAU führen. Nämlich dann, wenn ein kleines Kind das Opfer wird”. (Reader_NOZ) | "Okay, let's wait, this will take care of itself. It picks up speed on its own and will eventually lead to a super-GAU. Namely, when a small child becomes a victim". (Reader_NOZ) |
| Q12 | *"Wie man oft hören kann sind ja diese von ihnen beschriebenen Ängste vorhanden, ob nun zu Recht oder nicht spielt keine Rolle. Ich frage mich warum wir uns, oder denen die Angst haben, das denn antun wollen nur um ein paar rechthaberischen Wolfromantikern Recht zu geben. Angst ist nichts Schönes, und man sollte sich da nicht drüber lustig machen”. (Reader_NOZ)* | *"As you often hear, the fears you describe exist, whether justifiably or not is irrelevant. I ask myself why we want to do this to ourselves, or to those who are afraid, just to prove a few opinionated wolf romantics right. Fear is not a nice thing, and one should not make fun of it". (Reader_NOZ)* |
| Q13 | *"Das Risiko, von einem Wolf angefallen zu werden, gilt als extrem gering. In Europa gab es seit 40 Jahren keinen tödlichen Wolfsangriff auf Menschen, sieht man von einer Attacke im Gehege eines schwedischen Zoos ab, bei dem eine Wärterin starb”. (Artikel Spiegel)* | *"The risk of being attacked by a wolf is considered extremely low. There has not been a fatal wolf attack on humans in Europe for 40 years, apart from an attack in the enclosure of a Swedish zoo in which a keeper died". (Article_Spiegel)* |
| Q14 | *"Aber die Gefahr ist doch wohl eher theoretischer Natur. Wenn ich mich davor fürchten würde, dürfte ich überhaupt nicht raus in die Natur fahren, die Teilnahme am Straßenverkehr ist in jedem Fall um ein Vielfaches höher. Dazu kommen dann z.B. noch durch Zecken übertragene schwere Krankheiten, freilaufende gefährliche Hunde, renitente Wildschweine, kriminelle Mitmenschen, herabstürzende Äste…”* (Reader_NOZ) | *"But the danger is more theoretical. If I were afraid of it, I wouldn't be allowed to go out into nature at all; in any case, driving on the road is much more dangerous. Then there are, for example, serious diseases transmitted by ticks, dangerous dogs running loose, unruly wild boars, criminal fellow human beings, falling branches". (Reader_NOZ)* |
| *Q15:* | *"Die Tiere gehören zum Neißeland und zu dessen besonderer Naturlandschaft. Als solches vermarkten wir sie auch”.* (Article_Lausitzer Rundschau) | *"The animals belong to the Neisseland and its special natural landscape. We also market them as such*". (Article_Lausitzer Rundschau) |
| *Q16* | *"Das eigentliche Problem sind, dass die "urbane Elite" in einem "anderen Land" lebt, als die Deutschen auf dem Lande. Willkommenskultur für ein Raubtier, mich erstaunt, dass nicht an die armen Schafe und anderen Nutztiere gedacht wird, die brutal getötet werden und dazu noch psychisch durch den Jagddruck leiden!!!“ (Reader_FAZ)* | *"The real problem is that the ‘urban elite’ live in a ‘different country’ than the Germans in the countryside. ‘Willkommenskultur’ for a predator, I am amazed that no thought is given to the poor sheep and other farm animals that are brutally killed and, in addition, suffer psychologically due to the pressure of hunting!!!" (Reader_FAZ)* |
| *Q17* | *"Daher entfallen auf den ländlichen Raum viel weniger Wahlkreise, folglich ergibt sich dadurch eine Dominanz der Städter mit ihren verschrobenen Meinungen über Landwirtschaft und die Landbevölkerung”.* (Reader_FAZ) | *"Therefore, rural areas account for far fewer constituencies; consequently, this results in a dominance of urbanites with their ill-informed opinions about agriculture and the rural population". (Reader_FAZ)* |
| *Q18* | *"Da im ländlichen Raum die Siedlungsdichte gering ist, können die städtischen Mehrheiten in den Parlamenten mit der Landbevölkerung Schlitten fahren”.* (Reader_FAZ) | *"Since the density of settlement is low in rural areas, urban majorities in parliament can go sledging with the rural population." (Reader_FAZ)* |
| *Q19* | *"Die Menschen in den Wolfsgebieten leiden heute schon unter massiven Einschränkungen Ihrer Lebensqualität […] Es ist wohlfeil, wenn naturbewegte Staedter für den Wolf sind, dem sie niemals in ihrem Leben begegnen werden!” (Reader_FAZ)* | *"The people in the areas where wolves live are already suffering from massive restrictions on their quality of life. [...] It's unfair for nature-minded urban people to be in favour of the wolf, which they will never encounter in their lives!" (Reader_FAZ)* |
| *Q20* | *"…. wir werden von den Naturschutzbehörden mit Wolfspropaganda zugeschüttet. Aber wenn betroffene Weidetierhalter die grausame Wirklichkeit zeigen, schreitet die Staatsgewalt ein. Was für eine Anmaßung gegenüber der Landbevölkerung!” (Reader_TopAgrar)* | *"We are bombarded with wolf propaganda by the nature conservation authorities. But when concerned graziers show the cruel reality, the state authorities intervene. What a complete dismissal of the rural population!" (Reader_TopAgrar)* |
| *Q21* | *"Wer von den Leuten hier lebt eigentlich in einer Situation wo ein Wolfsangriff überhaupt ein Thema wäre? Das Risiko und die Furcht Anderer herunterzuspielen nur weil es für einen selber Bequem ist, ist nämlich auch nicht gerade eine Tugend”. (Reader_Spiegel)* | *"Who of the people here actually lives in a situation where a wolf attack would be an issue at all? Playing down the risk and the fear of others just because it is convenient for oneself is not exactly a virtue either". (Reader_Spiegel)* |
| *Q22* | *"Als "der mit dem Wolf tanzte" lernten wir alle, dass Wölfe liebe Tiere sind - und Indianer bessere Menschen. Seither sind beide "mega-in", vor allem in städtischen Kreisen, die praktisch nicht viel mit beiden zu tun haben, außer, wenn sie im Urlaub die Reservationen fluten, um ‘echte indianische Spiritualität’ zu konsumieren”.*  *(Reader_Spiegel)* | *"When Dances with Wolves came out, we all learned that wolves are dear animals—and Native Americans are better people. Since then, both have been ‘mega-in’, especially in urban circles that have practically nothing to do with either, except when they flood the reservations on holiday to consume ‘real Indian spirituality.’" (Reader_Spiegel)* |
| Q23: | "Mit dem Wolf ist es wie mit dem Windrad: Der Landbewohner zahlt für den Traum des Städters vom ‘intakten Ökosystem’ ebenso wie für den von der "sauberen Energie’”. (Reader_FAZ) | "The wolf is like the wind turbine: the rural dweller pays for the city dweller's dream of an ‘intact ecosystem’ as well as for that of ‘clean energy.’" (Reader_FAZ) |
| Q24 | *"[Sie] fühlen sich als Landwirte in die Defensive gedrängt, unter permanentem Rechtfertigungsdruck gegenüber Menschen, die ihre Arbeitstage im Büro verbringen und samstags in der Markthalle Bio kaufen”. (Article_FAZ)* | *"[They] feel put on the defensive as farmers, under permanent pressure to justify themselves to people who spend their working days in the office and buy organic food in the market hall on Saturdays". (Article_FAZ)* |
| Q25 | *"Entsprechend romantisiert wird der Wolf wahrgenommen. Vor allem natürlich von denen, die Natur vorwiegend aus den Medien oder dem Urlaub kennen. Was die unter Natur verstehen, hat mit Natur meist nicht viel zu tun - eher mit Fantasy..". (Reader_Spiegel)* | *"The wolf is perceived in a correspondingly romanticized way, above all, of course, by those who know nature mainly from the media or from holidays. What they understand as nature usually doesn't have much to do with nature—more with fantasy". (Reader_Spiegel)* |
| Q26 | *"Von daher starker Verdacht, dass die ahnunngslosen Stadtkinder der Landbevölkerung ihre Wünsche aufs Auge drücken. Da wird ein Raubtier (auch hier in den Kommentaren) harmlos—von Leuten, die auf dem Ferienbauernhof erschrecken, wenn eine Kuh zur Abwechslung einmal niest”. (Reader_FAZ)* | *"Hence the strong suspicion that the clueless city children are imposing their wishes on the rural population. There, a predator (also here in the comments) becomes harmless—and this from people who get scared while on holiday at a farm when a cow sneezes". (Reader_FAZ)* |
| Q27 | *"Was also passiert mit dem Wald, unserer Freiheit, der psychischen Gesundheit von uns allen jenseits der Latte Machiato Kultur im urbanen Dschungel, wo ‘Wolfsmetropolisten’ durch die Existenz von Wildnis ökologisch, sozial beruhigt sind? Die Natur, wie wir sie kennen, wird beerdigt. in Hamburg oder Berlin stört das nicht”. (Reader_FAZ)* | *"So, what happens to the forest, our freedom, the mental health of us all beyond the latte macchiato culture in the urban jungle where ‘wolf meteorologists’ are ecologically and socially pacified by the existence of wilderness? Nature as we know it is being buried. In Hamburg or Berlin, this doesn't bother us". (Reader_FAZ)* |
| Q28 | "Offensichtlich sind die Probleme in der Fläche noch nicht im *fernen Berlin* angekommen […] Allerdings wird eines deutlich: Wir dürfen uns nicht auf den Bund verlassen, denn dann sind wir verlassen, zumindest wenn es um die Frage des praktischen Umgangs mit Wolf und Biber geht”. (Artikel_FAZ) | "Obviously, the problems in the area have not yet reached distant Berlin [...] However, one thing becomes clear: we must not rely on the federal government, because then we are abandoned, at least when it comes to the question of how to deal with wolves and beavers in practice". (Article_FAZ) |
| Q29 | *"Bevor man zum Beispiel andere Gegenden Deutschlands als 'Wolfserwartungsland' ausweist, sollte man vielleicht wirklich mit einem Wolfsrudel im Berliner Regierungsviertel ein Zeichen setzen". (Reader_FAZ)* | *"For example, before designating other areas of Germany as 'wolf country', perhaps one should really set an example with a pack of wolves in Berlin's government quarter."(Reader_FAZ)* |
| Q30 | *"Unsere etablierten Parteien müssen sich über die Abkehr nicht wundern. Alles Weltfremd”. (Reader_TopAgrar)* | *"Our established parties need not be surprised at the abandonment. It’s all unworldly". (Reader_TopAgrar)* |
| Q31 | *"Welche Partei sie wählen, ob sie überhaupt wählen – solche Fragen beantworten die beiden mit einem Schweigen und indem sie sich vielsagende Blicke zuwerfen. Klar wird: Von den Berufspolitikern in Berlin und Brüssel halten sie wenig”. (Article_FAZ)* | *"Which party they vote for, whether they vote at all—the two answer such questions with silence and by casting meaningful glances at each other. It becomes clear: They think little of the professional politicians in Berlin and Brussels". (Article_FAZ)* |
| Q32 | *"Jeder kann sich überlegen, wen er bei einem so hanebüchenen Bullshit bei der kommenden Europawahl wählt. Es wird immer schlimmer mit dem Verein. Der Brexit ist bekloppt, aber manchmal versteht man dei Beweggründe der Briten”.*  (Reader_TopAgrar) | *“Everyone thinks about who they will vote for in the coming European elections with such absurd bullshit. It's getting worse and worse with the club. Brexit is stupid, but sometimes you understand the motives of the Brits*". (Reader_TopAgrar) |
| Q33 | *"Nun plagen ländliche Regionen in Sachsen und Brandenburg durchaus auch andere Probleme als der Wolf. Noch immer ziehen viele junge Menschen weg, es herrscht chronischer Ärztemangel, die Anbindung an den öffentlichen Verkehr ist miserabel, an vielen Stellen ist das Internet sehr langsam. Wahlkampf aber wird viel und gern mit dem Wolf gemacht”.* (Artikel_Spiegel) | *"Now, rural regions in Saxony and Brandenburg are plagued by problems other than the wolf. Many young people are still moving away, there is a chronic shortage of doctors, public transport connections are miserable, and the internet is very slow in many places. Election campaigns, however, are often gladly concerned with the wolf". (Article_Spiegel)* |
| *Q34* | *"Es ist seltsam, immer wenn Personen zum Thema Wolf befragt werden sind es überwiegend Wolfsbefürworter die dann auch in der Presse mitgeteilt werden. Und es ist seltsam, dass Kommentare gegen den Wolf sofort gemeldet und entfernt werden. Und wenn ein Kommentar gegen den Wolf erscheint, fallen die Befürworter wie die Wölfe über diese Person her und zereißen diesen wie die Wölfe die Schafe, Rinder und Pferde zereißen mit zum Teil sehr dummen Kommentaren”.* (Reader_NOZ) | *"It is strange that whenever people are interviewed on the subject of wolves, it is predominantly wolf supporters who are then also reported in the press. And it is strange that comments against the wolf are immediately reported and removed. And when a comment against the wolf appears, opponents fall like wolves upon this person and tear him apart, like wolves tear sheep, cattle and horses apart, with sometimes very stupid comments". (Reader_NOZ)* |
| *Q35* | *"Im Landkreis Vechta trauen sich die Schäfer schon garnicht mehr ihre Verluste zu melden, da sie sonst Drohungen erhalten. Einseitige Berichterstattung in der Lokalpresse”.* (Reader_FAZ) | *"In the district of Vechta, shepherds no longer dare to report their losses because they receive threats. There is one-sided reporting in the local press*". (Reader_FAZ) |
| *Q36* | *"Schon einmal hat uns eine unüberlegte "Willkommenskultur"...in eine Lage gebracht, deren Auswirkungen wir jetzt tagtäglich den Medien entnehmen können bzw. am eigenen Leibe erfahren müssen”.* (Reader_FAZ) | *"Once before, an ill-considered ‘welcome culture’... brought us into a situation whose effects we can now see every day in the media or have to experience first-hand". (Reader_FAZ)* |
| Q37 | *"Die AfD zieht seit längerer Zeit offen Vergleiche zwischen dem Vordringen der Wölfe nach Deutschland und der Flüchtlingsbewegung”.* (Article_FAZ) | *"The AFD has been openly drawing comparisons between the advance of wolves into Germany and the refugee movement for some time". (Article_FAZ)* |
| Q38 | *"Die AfD fordert eine Obergrenze – und meint in diesem Fall nicht Flüchtlinge, sondern Wölfe. Das Beispiel zeigt: Mit dem Raubtier wird Politik gemacht, erst recht vor den Wahlen in Ostdeutschland”. (Article_*Spiegel) | *"The AFD is calling for an upper limit—and in this case it doesn't mean refugees, but wolves. This example shows that politics are driven by the predator, even more so before the elections in East Germany". (Article_Spiegel)* |
| *Q39* | *"Alles und jedes in diesem Land ist transparent, nachvollziehbar und muss begründet werden, lediglich wenn es um Wolfsrisse geht, dann werden die Details zur geheimen Verschlusssache erklärt. Mir leuchtet das nicht ein. Vielleicht gilt aber auch hier das inzwischen geflügelte Wort des ehemaligen Bundesinnenministers: Ein Teil dieser Antworten würde die Bevölkerung verunsichern”. (Article_FAZ)* | *"Everything and anything in this country is transparent, comprehensible and must be justified, but when it comes to wolf attacks, the details are declared a secret. That doesn't make sense to me. But perhaps the former federal minister of the interior's now familiar saying also applies here: Some of these answers would unsettle the population". (Article_FAZ)* |
| *Q40* | *"Es ist eine Menge Gift in der Debatte, von beiden Seiten”. (Artikel_Spiegel)* | *"There is a lot of venom in the debate, from both sides". (Article_Spiegel)* |
| *Q41* | *"Es wird Zeit, dass wir sie mit der Waffe in der Hand verteidigen”. (Reader_TopAgrar)* | *"It's time we defended them with a gun in hand". (Reader_TopAgrar)* |
| *Q42* | *"Wenn der Staat kein Verständnis für die Belange seiner Bürger hat, helfen die sich selbst.  Nicht mit unbezahlbarem Weideschutz-Zaun oder hochaggressiven Kampfhunden, sondern billig und einfach mit Gift. Eine buchstänlich todsichere Wolfsabwehr, die Täterschaft ist nicht nachweisbar, der Erfolg garantiert”. (Reader_FAZ)* | *"If the state has no understanding of the concerns of its citizens, they must help themselves.*  *Not with unaffordable pasture protection fences or highly aggressive attack dogs, but cheaply and simply with poison. A literally sure-fire wolf defence, the perpetration cannot be proven, the success is guaranteed". (Reader_FAZ)* |
| *Q43* | *" SSS Schießen- Scharren- Schweigen". (Reader_TopAgrar)* | *"SSS shoot—scrape—silence" (Reader_TopAgrar)* |
| *Q44* | *"Die beste Wolfsregulierung wäre die scharfe Bejagung”. (Reader_TopAgrar)* | *"The best wolf regulation would be sharp hunting". (Reader_TopAgrar)* |
| *Q45* | *"Den Wolf abschießen! Das ist nämlich die beste und gleichzeitig auch billigste Prävention!!!! Nicht umsonst wurde er bei uns bis auf NULL reduziert”. (Reader_TopAgrar)* | *"Shoot the wolf! Because that is the best and at the same time also the cheapest prevention!!!! Not for nothing was the population reduced to ZERO in our country". (Reader_TopAgrar)* |
| *Q46* | *"Dabei zeigt sich doch, dass die Politik handelt, in dem ein Wolf abgeschossen werden soll, der die als wolfssicher geltenden Schutzzäune zu überwinden gelernt hat. Das ist grundsätzlich das richtige Vorgehen, um die Akzeptanz der Wölfe zu erhalten und den Schaden an Nutztieren zu begrenzen. Mit weiter wachsender Wolfspopulation wird ohnehin in ein paar Jahren ein Zustand erreicht sein, bei dem nicht nur über den Abschuss auffälliger Einzeltiere, sondern über eine Bestandsregulation insgesamt diskutiert werden wird. Wenn die Bestandsentwicklung und die Auffüllung noch wolfsfreier Verbreitungslücken in Deutschland und seinen Nachbarländern so weiter verläuft, habe ich dagegen auch nichts Grundlegendes einzuwenden”. (Reader_NOZ)* | *"This shows that politics is taking action by shooting a wolf that has learned to break through the protective fences that are considered wolf-proof. In principle, this is the right course of action to maintain the acceptance of wolves and to limit the damage to farm animals. As the wolf population continues to grow, a situation will be reached in a few years anyway in which not only the shooting of conspicuous individual animals but also population regulation as a whole will be discussed. If the population development and the filling of still wolf-free areas in Germany and its neighbouring countries continues in this way, I have no fundamental objections to this". (Reader_NOZ)* |
| *Q47* | *"Es gehe darum, eine Balance zwischen den berechtigten Sorgen der Weidetierhalter auf der einen Seite und der an sich wünschenswerten Wiederansiedlung des Wolfes herzustellen”. (Article_FAZ)* | *“It is a matter of striking a balance between the justified concerns of grazing livestock farmers on the one hand and the desirable reintroduction of the wolf on the other". (Article_FAZ)* |
| *Q48* | ***“****Meine Fraktion fordert seit Jahren die Aufnahme des Wolfes in das Jagdrecht, die Festlegung von Obergrenzen, die Festlegung von wolfsfreien Zonen, Möglichkeiten der rechtskonformen Entnahme, die Veröffentlichung der Ergebnisse der Rissgutachten, die klare Definition von Problemwölfen und -rudeln sowie die hundertprozentige Entschädigung von Nutztierhaltern”. (Artikel_FAZ)* | *"For years, my parliamentary representatives have been demanding the inclusion of the wolf in hunting law, the setting of upper limits, the definition of wolf-free zones, possibilities for legally compliant removal, the publication of the results of the expert's reports on wolves, the clear definition of problem wolves and packs as well as one hundred percent compensation for livestock owners". (Article_FAZ)* |
